# An Improved Polymerase Cross-Linking Spiral Reaction Assay for Rapid Diagnostic of Canine Parvovirus 2 Infection

**DOI:** 10.3389/fvets.2020.571629

**Published:** 2020-10-30

**Authors:** Xin Xu, Xueyu Wang, Wen Hu, Qianqian Wu, Lunguang Yao, Yunchao Kan, Jun Ji, Yingzuo Bi

**Affiliations:** ^1^Henan Provincial Engineering and Technology Center of Health Products for Livestock and Poultry, Nanyang Normal University, Nanyang, China; ^2^Collaborative Innovation Center of Water Security for Water Source Region of Mid-line of South-to-North Diversion Project of Henan Province, School of Agricultural Engineering, Nanyang Normal University, Nanyang, China; ^3^College of Animal Science, South China Agricultural University, Guangzhou, China

**Keywords:** canine parvovirus, polymerase cross-linked spiral reaction, detection method, simplicity, visible detection

## Abstract

With increasing complications of canine parvovirus infection cases, disease diagnosis and treatment have become more difficult. In this study, specificity primers for the conserved region of the VP2 gene of canine parvovirus 2 (CPV-2) were synthesized and evaluated. An improved polymerase cross-linking spiral reaction (PCLSR) method for early and rapid diagnosis of CPV-2 was established. The results showed that the amplification reaction was optimal when run at 62°C for 50 min and could be used to detect CPV-2 without any cross-reactions with other pathogens of canine infectious diseases. Reaction results were directly judged by the naked eyes, with the positive amplification tube shown as luminous yellow and the negative tube as bright purple. Compared with the previously reported polymerase spiral reaction (PSR) method for CPV-2 detection, this reaction was performed using improved primer pairs and a better dye identification method (using an indicator comprising phenol red and cresol red). The detection limit of PCLSR was 3.9 × 10^1^ copies using gel electrophoresis or a visible dye. The positive rate of 132 clinical samples was 42.42%, which was identically the same as that of the PSR method and slightly higher than that of the colloidal gold strip method (39.39%). The newly developed CPV-PCLSR assay shows the advantage of rapid visualization of results and offers a convenient and rapid method for early CPV-2 diagnosis with higher sensitivity and specificity than the established methods.

## Introduction

Canine parvovirus 2 (CPV-2) belongs to the species *Carnivore protoparvovirus* 1, genus *Parvovirus* of the family *Parvoviridae*. It is a single-stranded linear DNA virus, which was first isolated from infected dogs in the United States in 1978 and subsequently spread worldwide (1). In China, CPV-2 was first isolated and reported in 1983, and infections have been regularly reported ever since. The morbidity and mortality rates of CPV-2 infection are as high as 70% (2). Globally, CPV-2 is one of the most threatening pathogens for domestic and wild dogs. Meanwhile, the symptoms caused by CPV-2 infection in diseased dogs are similar to those of infections by other enteroviruses and bacteria, which is a challenge for clinical diagnostics (3). Simultaneously, mutations of the virus gene have posed many difficulties in disease diagnosis and treatment, such as the emergence of new strains, concomitant infections, and atypical clinical symptoms. Therefore, timely and accurate diagnosis is paramount to offer the most appropriate interventions and treatments for the rehabilitation of infected dogs (4).

Recently, with the rapid increase in pet ownership, coinfections of CPV-2 with canine coronavirus (CCoV) and canine adenovirus (CAV), which also cause canine viral enteritis, even reached up to 55.6% (5, 6). Furthermore, the cure rate of CPV-2 in the early stage of infection is significantly higher than that in the late stage of infection; therefore, pet hospitals and primary veterinary clinics must urgently establish efficient, rapid, and specific early diagnostic methods for sick dogs. At present, the diagnostic methods for CPV-2 are mainly divided into molecular and immunological diagnostic methods. The former methods focus on viral nucleic acids and include conventional PCR, real-time PCR, loop-mediated isothermal amplification (LAMP), and recombinase polymerase amplification (RPA), among others (6–9). Immunological diagnostic methods are mainly directed against antigens and antibodies produced following immunization and include hemagglutination test, hemagglutination inhibition test, and ELISA, among others (10, 11). Furthermore, gold nanoparticle-based immunochromatographic strip test based on a combination of mAb and pAb is an acceptable alternative for the diagnosis of CPV-2 infection (12). Although immunological diagnosis is cost effective and easy to operate, it has disadvantages of being time consuming and having low sensitivity and specificity (13). In contrast, highly sensitive PCR-related methods cannot be widely applied because of their complex operation and requirement of expensive instruments for thermal cycling and gel electrophoresis (6). The LAMP assay for CPV-2 detection requires strict primer coordination for targeting six sequence regions and a more conserved sequence (14). Furthermore, although the RPA method requires only one primer pair to accomplish detection, its reaction products cannot be directly determined by the naked eye (9).

Detection methods based on isothermal amplification of nucleic acids, which can rapidly synthesize large amounts of DNA without any specific requirements for precision instruments, have been widely used. By improving the conventional molecular diagnostic methods, a highly sensitive and specific polymerase spiral reaction (PSR) based on standard PCR and LAMP was developed to combine their advantages (15). PSR detection methods have been used for CPV-2 (16). Meanwhile, product formation is accompanied by high levels of pyrophosphate ion by-product, leading to a change in pH. Therefore, a pH-sensitive dye can be used to detect the product of the reaction with the naked eye (17). Furthermore, the polymerase cross-linking spiral reaction (PCLSR) method uses a set of specially designed primers, two external helix primers, and an additional special cross-linked primer has been developed for rapid detection of African swine fever virus (18). Based on specificity, the method offers an accelerated reaction rate, simple operation, and good stability. Therefore, to offer a novel alternative assay for CPV-2 detection, PCLSR applications were explored and optimized in this study.

## Materials and Methods

### Sample Collection and Nucleic Acid Extraction

All canine infectious disease samples are stored at the Henan Provincial Engineering Laboratory of Insects Bio-reactor, Nanyang Normal University, China, and included CPV-2 (commercial vaccine strain, Vanguard Plus 5, Pfizer Inc., Lincoln, NE, USA); three CPV-2 variants (CPV-2a, MK518002, CPV-2b, MK517983, and CPV-2c, MK517966); and canine distemper virus (CDV)-, canine coronavirus (CCoV)-, canine circovirus (CCV)-, canine adenovirus-1 (CAV-1)-, and canine adenovirus-2 (CAV-2)-positive samples; and 59 CPV-2 isolates were determined by virus isolation and VP2 sequencing that were collected from 2016 to 2019 (CPV-2a, *n* = 17; CPV-2b, *n* = 12; and CPV-2c, *n* = 30). A total of 132 clinical samples were collected from dogs with acute or mild gastroenteritis provided by the outpatient departments of pet hospitals and dog farms from 2018 to 2019 in Henan, Hubei, Anhui, and Jiangsu provinces, China. The research protocols for sample collection protocols were approved by the dog's owner. Viral genomic DNA/RNA was extracted from the feces of suspected animals using the EasyPure Viral DNA/RNA Kit (TransGen Biotech, Beijing, China) with nucleic acid-binding potential of matrix material supplied in spin columns as per the manufacturer's instructions. Viral DNA or cDNA synthesized through first-strand synthesis with the RevertAid Minus First Strand cDNA Synthesis kit (Fermentas Inc., Hanover, MD) was used for testing.

### Polymerase Cross-Linking Spiral Reaction Primers

Four primers were designed to detect the VP2 conserved region through multiple-sequence alignment of the published CPV-2 gene sequences available in GenBank, NCBI (up to November 2019) using Clustal W in MEGA 7.0. External spiral primer pairs were used to complete spiral amplification, including the external spiral forward primer SF and external spiral reverse primer SR; cross-linking primers (CLF and CLR) were also used to facilitate chain replacement and amplification to accelerate the reaction process. The scheme explaining PCLSR assay and the target positions of the primers are presented in Supplementary Figure 1. PCLSR primers are listed in Table 1.

**Table 1:** Primer information used in this study.

**Primer**	**Sequence (5^**′**^-3^**′**^)**	Position*
External spiral primer	SF: *acgaattcgtacatagaagtatag*-tttgaggcgtctacacaaggg	1,033–1,053
	SR: *gatatgaagatacatgcttaagca*-ggtgtttctcctgttgtggtagt	1,162–1,183
Cross-linking primer	CLF: atctgtttgcgctcccccccgt	1,080–1,101
	CLR: agatggtgatccaagatatgca	1,116–1,137

*The sequences in italics are upstream and downstream reverse complement sequences derived from the literature and are modified sequences from plant source transformation sequences (18)*.

* *The primer position is based on the sequence of the HB1 strain, GenBank accession number: GU392236*.

### Development of Polymerase Cross-Linking Spiral Reaction Assay: Optimization of Reaction Temperature and Time

PCLSR was performed in a 25 μl liquid reaction mixture containing 1.6 mM each of SF and SR, 0.8 mM CLF and CLR, 1.2 mM dNTPs (TaKaRa Biotech Corporation, Dalian, China), 8 U of *Bst* 2.0 DNA polymerase (New England Biolabs, Ipswich, MA, USA), 10 mM (NH_4_)_2_SO_4_, 50 mM KCl, 8 mM MgSO_4_, 0.1% v/v Tween-20, 1 μl of template DNA of the commercial vaccine strain of CPV-2, and 1 μl of color indicator (0.025 mM phenol red and 0.08 mM cresol red) (Solarbio, Beijing, China) for the visualization of products; ddH_2_O was used to make up the volume to 25 μl. Reaction mixture without a template was used as a negative control for comparison. To avoid cross-contamination and residual contamination caused by the volatilization of reaction products, 30 μl of inert liquid mineral oil was added to each tube after configuration of the reaction to seal the reaction liquid. Optimum reaction conditions were standardized as follows: temperature ranging from 60 to 65°C and reaction time ranging from 30 to 55 min (Supplementary Table 1). After completion of the reaction, color change was observed under natural light by three researchers at least. Simultaneously, 5 μl of the product was subjected to 2% agarose gel electrophoresis (0.5 μg/ml ethidium bromide) for detection.

### Sensitivity of Polymerase Cross-Linking Spiral Reaction

The VP2 gene (1,755 bp) from the commercial vaccine strain of CPV-2 was amplified and cloned into a commercial clone vector pMD 18-T (TaKaRa Biotech Corporation, Dalian, China) to construct a standard plasmid (CPV-2-VP2). The standard plasmid (CPV-2-VP2) was diluted 10-fold to obtain 3.9 copies. The analytical sensitivity of the newly developed PCLSR assay was compared with that of other molecular techniques, including quantitative real-time PCR (qPCR) and PSR assay. The PSR mixture (25 μl) included 8 U of *Bst* 2.0 DNA polymerase, and PSR was performed using a water bath at 60°C for 75 min, as described previously (17). The qPCR reaction was performed on a CFX96 Touch detection system (Bio-Rad, Hercules, CA, USA) in a total volume of 25 μl containing 12.5 μl of HotStarTaq Master Mix Kit (Qiagen, Hilden, Germany), primer pairs (10 pmol), and probe (5 pmol) as described previously (19). The limits of detection of PCLSR, PSR, and qPCR were determined using 10-fold dilutions. At the end of the reaction, gel electrophoretic and colorimetric analyses were performed to determine the sensitivity of the methods.

### Specificity of Polymerase Cross-Linking Spiral Reaction

Specificity of PCLSR was determined using other common canine disease samples (CDV, CCoV, CCV, CAV-1, and CAV-2) and three CPV-2 variants (CPV-2a, CPV-2b, and CPV-2c); CPV-2 DNA (commercial vaccine strain) was used as a positive control. Optimized reaction conditions were used to determine cross-reaction with other canine infectious disease pathogens.

### Clinical Testing

The optimized PCLSR assay was used to assess 59 CPV-2 isolates and 132 samples of suspected CPV-2 infection. Simultaneously, samples were examined through PSR and colloidal gold strip methods (BioNote Rapid Test Kit, Hwaseong-si, Korea), and positive rates with different detection methods were calculated and compared using qPCR as a gold standard. The samples tested positive for PCLSR but negative for other detection methods were further screened for the presence of CPV-2 DNA by PCR using a 20 μl reaction mixture containing a DNA template (products of PCLSR reaction), 10 pmol primers (SF and SR), and Ex-Taq DNA polymerase (TaKaRa Biotech Corporation, Dalian, China). The obtained amplicons were subsequently sequenced (Syn-Biotechnology, Suzhou, China).

The degree of agreement between PCLSR and qPCR test results was measured by kappa value (κ) (20). All statistical analyses were performed using IBM SPSS Statistics, version 25 (IBM Corporation, New York, USA).

## Results

### Optimum Reaction Temperature and Time for Canine Parvovirus 2 Diagnosis by Polymerase Cross-Linking Spiral Reaction

**Figure 1:**
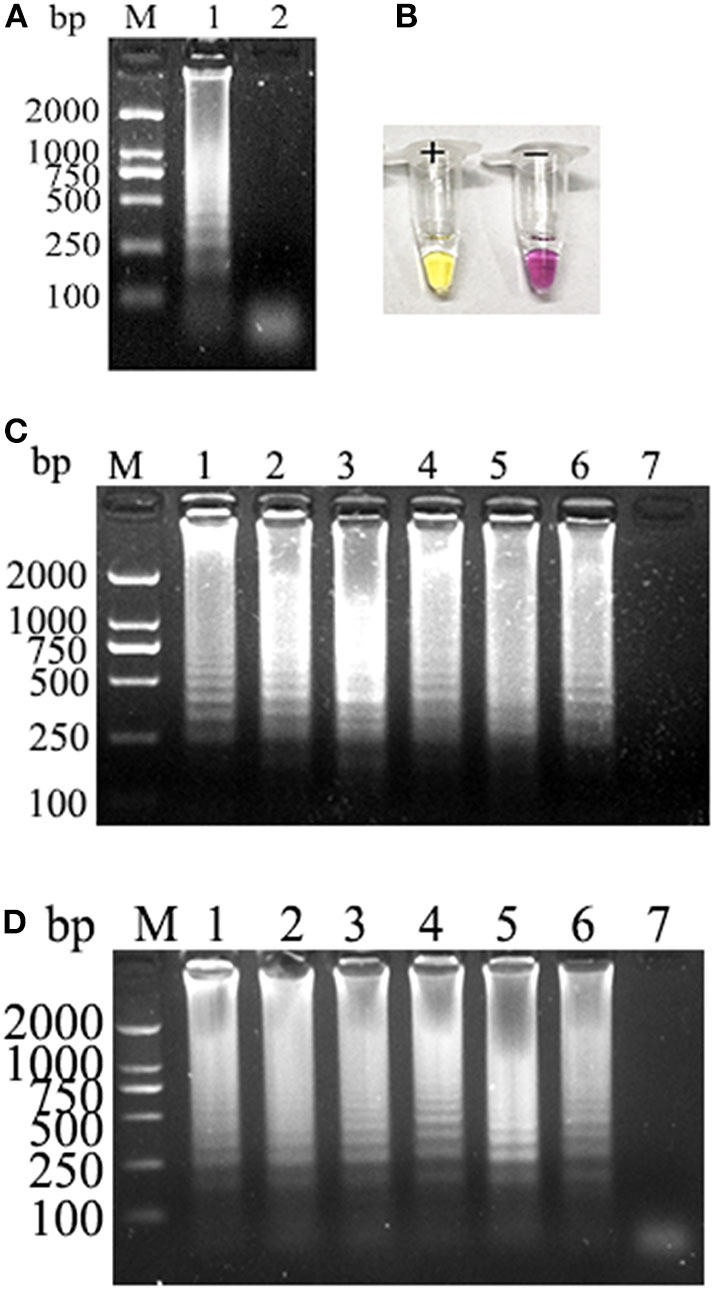
**(A)** Polymerase cross-linking spiral reaction (PCLSR) amplification in the conserved region of the canine parvovirus (CPV) VP2 gene. **(B)** Color change of PCLSR amplification products. M, 2,000 DNA ladder marker; 1, PCLSR amplified products; 2, negative control. **(C)** PCLSR amplification results at different temperatures. M, 2,000 DNA ladder marker; 1: 60°C; 2: 61°C; 3: 62°C; 4: 63°C; 5: 64°C; 6: 65°C; 7: negative control. **(D)** PCLSR amplification results at different reaction times. M, 2,000 DNA ladder marker; 1: 30 min; 2: 35 min; 3: 40 min; 4: 45 min; 5: 50 min; 6: 55 min; 7: negative control.

Electrophoretic bands showed a ladder-like distribution, and the negative control showed the primer dimer band alone, which is consistent with the theoretical results (Figure 1A). At the end of the reaction, the positive amplifier tube appears bright yellow, and the negative tube remains bright purple (Figure 1B). Electrophoresis results showed that PCLSR could be performed at all six tested temperatures (60, 61, 62, 63, 64, and 65°C) and that the amplification efficiency showed no obvious differences. Reaction efficiency was the highest at 62°C, which was regarded as the best reaction temperature (Figure 1C). Moreover, amplification time was optimized between 30 and 55 min; the results became increasingly obvious over time, with the best result obtained at 50 min (Figure 1D). Assay data used for optimum reaction temperature and time for CPV-2 diagnosis by PCLSR are displayed in detail in Supplementary Table 1. Based on these results, the optimum PCLSR conditions were confirmed as 62°C and 50 min.

### Sensitivity of Polymerase Cross-Linking Spiral Reaction

**Figure 2:**
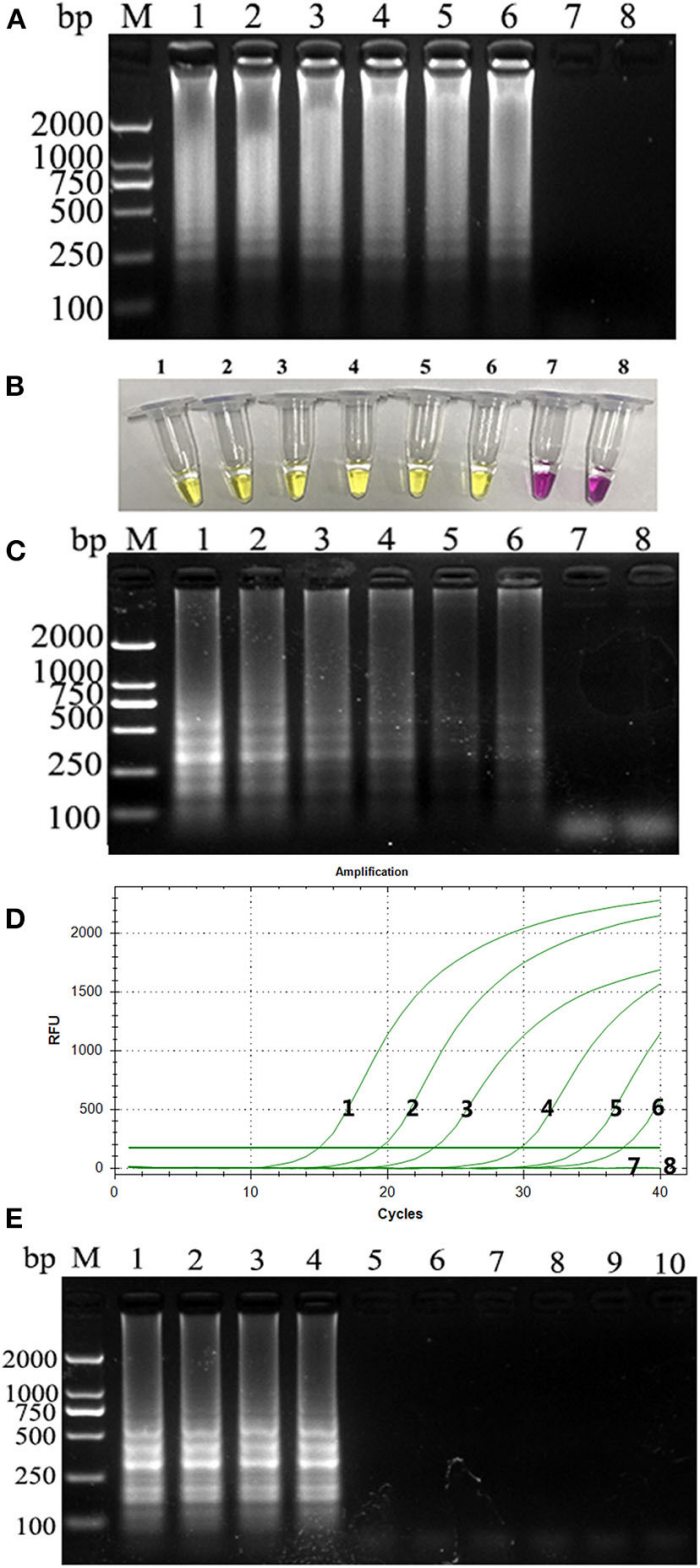
**(A)** Sensitivity of the CPV-PCLSR amplification; **(B)** results of indicator discoloration; **(C)** PSR amplification; **(D)** qPCR. M, 2,000 DNA ladder marker; 1–7, DNA template with 3.9 × 10^6^ to 10^0^ copies; 8, negative control; **(E)** specificity of the CPV-PCLSR amplification. M, 2,000 DNA ladder marker; 1, canine parvovirus 2 (CPV-2) from vaccine; 2, CPV-2a; 3, CPV-2b; 4, CPV-2c; 5, CDV; 6, CCoV; 7, CCV; 8, Dogav-1; 9, Dogav-2; 10, negative control.

PCLSR was performed with diluted recombinant plasmid gradient. The detection limit of PCLSR, PSR, and qPCR was 3.9 × 10^1^ copies. Results of electrophoretic imaging analysis (Figures 2A,B,D) were consistent with the results of color change analysis (Figure 2C).

### Specificity of Polymerase Cross-Linking Spiral Reaction

Results of canine pathogen detection using PCLSR amplification are shown in Figure 2E. The designed primers did not react with other viral nucleic acids and showed a good specificity to effectively distinguish CPV-2 from other canine pathogens.

### Clinical Testing Results

All 59 CPV-2 isolates were detected positive by PCLSR, which was consistent with the results of qPCR, PSR, and colloidal gold strip test. Moreover, PCLSR, qPCR, PSR, and colloidal gold strip test were also used to detect 132 suspected clinical canine samples, and the results showed 56, 56, and 52 positive samples, respectively (Table 2). The positive detection rate of PCLSR and qPCR was 42.42%, and the coincidence rate was 100% with kappa value of 1; the positive detection rate of the colloidal gold strip test was 39.39%. After cross-checking data for all samples, it was found that the samples not detected through the colloidal gold strip test were from dogs presenting with mild clinical symptoms, which were presumed to be early stages of CPV-2 infection as low viral load decreases the sensitivity of immunological detection. The presence of CPV-2 DNA in the four samples was confirmed by PCR and sequencing. Results of clinical samples showed that the positive detection rate of PCLSR was identical to that of PSR, indicating high reliability of PCLSR.

**Table 2:** Comparison of quantitative real-time PCR (qPCR), polymerase cross-linking spiral reaction (PCLSR), polymerase spiral reaction (PSR), and colloidal gold strip for detection of canine parvovirus 2 (CPV-2) in clinical specimens.

**qPCR**	**Detection rate for assay**		
	**PCLSR**	**PSR**	**Colloidal gold strip**
Positive samples	56/56*	56/56	52/56
Negative samples	76/76	76/76	80/76

56/56* indicates detection numbers for each assay/the detection numbers according to qPCR; agreement of results between PCLSR and qPCR, κ = 1.

## Discussion

Mutations of CPV-2 have been continuously reported since its clinical detection, and variants such as CPV-2a, CPV-2b, and CPV-2c have appeared successively (21). New variants show markedly different antigenicities and gene sequences; therefore, the accuracy, specificity, and sensitivity of CPV-2 detection methods must be high. PSR isothermal amplification was invented by Liu et al. (15). By adding reverse complementary sequences with upstream and downstream primers, nucleic acids were amplified under isothermal conditions through primer binding, extension, unlinking, single rotation, and re-extension *via* the action of *Bst* 2.0 DNA polymerase. Only a single pair of primers and a single enzyme are required. The complex chain replacement reaction can be completed without the repeated addition of sequences. Compared with the established PSR assay, improved PCLSR requires an additional pair of cross-linked internal primers, which can further accelerate the reaction speed and increase the prominence of advantages of this method (18). Because PCLSR produces a large amount of pyrophosphates in the reaction process, a pH indicator was added to the reaction mixture, making the reaction result easy to observe without affecting amplification. Following amplification, discoloration can be directly observed under natural light without electrophoresis to judge the amplification efficiency. This method is more suitable for testing at the grassroots level and in the field and greatly improves the detection efficiency.

With advances in molecular technologies, novel assays for CPV-2 detection have been developed and established. Combined with lateral flow strips (LFSs) and magnetic purification, the PCR-LFS assay detect CPV-2 in 80 min with high sensitivity while omitting the electrophoresis step for analyzing results (22). The direct TaqMan real-time PCR method was also established to diagnose CPV-2 and CDV in dogs without the requirement of nucleic acid extraction (23). A simple-probe real-time PCR assay with duplex fluorescence melting curve analysis and multiplexed tandem PCR panel was established to detect CPV-2 and to identify its variants (23). These PCR-related methods are sensitive and accurate; however, they require a thermocycler, and CPV-PCLSR requires a water bath. Even though CPV-PCLSR cannot differentiate CPV-2 genotypes, it meets the basic clinical demand for detection. As an isothermal method, insulated isothermal polymerase chain reaction (iiPCR) was also developed for CPV-2 detection, which can perform detection in 1 h, but it requires the use of a specific device (POCKIT^TM^ Nucleic Acid Analyzer) to partly restrict the detection throughput as well as a fluorescent probe, which increases the cost of detection (24). Furthermore, visible and equipment-free RPA combined with LFS was developed to detect CPV-2 in 30 min; these assays can even be performed in a closed fist using body heat (25). LFS-RPA required a shorter detection time than CPV-PCLSR and can be performed in a closed fist using body heat without any equipment, making it an attractive and promising tool for rapid and convenient diagnosis of CPV infections; however, the reaction kit, probe, and LFS are costly. CPV-2 PCLSR requires approximately 50 min for detection compared with the previously reported CPV-2 PSR assay, which requires 75 min (16). On the other hand, CPV-PSR using SYBR Green I dye can satisfactorily reveal dissimilarities between positive and negative tubes; however, the dye must be added at the end of the reaction for the inhibitory effect. For the highly sensitive LFS-RPA and PSR assays, the operations needed for reactions using lateral flow strips or *via* the addition of SYBR Green I dye increase the process as well as could increase the risk of false-positive results due to aerosol contamination by amplified products. Therefore, CPV-PCLSR with the addition of pH-sensitive dye before the reaction could reduce aerosol contamination and remains a promising alternative for CPV-2 detection. For determining the results of detection based on color change is somewhat subjective, screening a better dye that induces more apparent color change and without inhibitory effect for amplification would be important for the development of the PCLSR assay.

In this study, based on the VP2 sequence of CPV-2 in GenBank, a highly conserved region was analyzed and selected for primer design, and a rapid detection for CPV-2 (CPV-PCLSR) was established. The results showed that the addition of CLF/CLR did not affect amplification but optimized the reaction time instead, even though they slightly increased the experimental cost. The operator can freely choose the conditions according to the experimental requirements. The established PCLSR detection method can be performed at a wide temperature range from 60 to 65°C; the clinical testing was completed at 62°C in a water bath, thus eliminating the need for a thermocycler and other precision equipment. Moreover, the accuracy of CPV-PCLSR in detecting clinical samples is superior to that of colloidal gold strip test. As previously reported, detection tests based on colloidal gold strip have been proven to be extremely sensitive compared with real-time PCR (23). Even though only four more samples tested positive using the PCLSR assay compared with the colloidal gold strip method, PCLSR demonstrated a 100% compliance rate with the reported qPCR and PSR assay. Meanwhile, the presence of CPV-2 DNA in the four samples was confirmed by the sequencing of the amplification products using PCR with primer pairs (SF and SR) and PCLSR products from the four samples as template, respectively. The possible explanation for the high positive detection rate of the colloidal gold strip method may have been due to the high viral load of the 52 positive samples (copy numbers in these samples were determined to be above 5.56 × 10^5^ copies). Thus, the established PCLSR assay shows certain promise as an early clinical diagnostic tool for the detection of CPV-2. Moreover, PCLSR assay using *Bst* 2.0 DNA polymerase would theoretically be less affected by potential inhibitors within the samples, which indicated that DNA extraction from samples could be omitted (26). Therefore, future studies to evaluate the sensitivity of the PCLSR without DNA extraction methods or using the boiling method for DNA extraction from canine samples may be required. If PCLSR results will be confirmed using these samples, this may further suggest the use of the PCLSR method under field conditions.

## Conclusion

In summary, CPV-PCLSR established in this study meets the requirements of rapid multidose differential diagnosis of CPV-2 as well as its accurate and early detection, thus helping in the effective prevention and control of disease spread and improving the cure rate of infected animals. The proposed assay offers a new tool for rapid diagnosis of CPV-2 infections.

## Data Availability Statement

The original contributions presented in the study are included in the article/Supplementary Material, further inquiries can be directed to the corresponding author/s.

## Ethics Statement

Sampling was not harmful to the dogs. The research protocols for sample collection protocols were approved by the dog's owner and the Animal Welfare and Ethics Committee of Nanyang Normal University (No. 14027).

## Author Contributions

JJ, QW, and YB designed the study. XX, JJ, XW, and WH performed the experiments. WH, LY, and YK analyzed the data. XW wrote the paper. All authors have read and approved the final manuscript.

## Conflict of Interest

The authors declare that the research was conducted in the absence of any commercial or financial relationships that could be construed as a potential conflict of interest.

## Supplementary Material

Supplementary Figure 1 **(A–C)** Schematic representation of the specific primers for PCLSR assay; **(D)** Schematic representation of the annealing positions of PCLSR primers within the CPV-2 VP2 gene.

Supplementary Table 1 Assay data used for optimum reaction temperature and time for CPV-2 diagnosis by PCLSR.

## References

[1] Appel MJ Scott FW Carmichael LE. Isolation and immunisation studies of a canine parco-like virus from dogs with haemorrhagic enteritis. Vet Rec. (1979) 105:156–9. 10.1136/vr.105.8.156 516347

[2] Hoelzer K Parrish CR. The emergence of parvoviruses of carnivores. Vet Res. (2010) 41:39. 10.1051/vetres/2010011 20152105 PMC2844231

[3] Battilani M Balboni A Ustulin M Giunti M Scagliarini A Prosperi S. Genetic complexity and multiple infections with more parvovirus species in naturally infected cats. Vet Res. (2011) 42:43. 10.1186/1297-9716-42-43 21366901 PMC3059301

[4] Miranda C Thompson G. Canine parvovirus: the Worldwide occurrence of antigenic variants. J Gen Virol. (2016) 97:2043–57. 10.1099/jgv.0.000540 27389721

[5] Perez R Calleros L Marandino A Sarute N Grecco S Blanc H. Phylogenetic and genome-wide deep-sequencing analyses of canine parvovirus reveal co-infection with field variants and emergence of a recent recombinant strain. PLoS ONE. (2014) 9:e111779. 10.1371/journal.pone.0111779 25365348 PMC4218814

[6] Deng X Zhang J Su J Liu H. A multiplex PCR method for the simultaneous detection of three viruses associated with canine viral enteric infections. Arch Virol. (2018) 163:2133–238. 10.1007/s00705-018-3828-4 29675651 PMC7086948

[7] Sun Y Cheng Y Lin P. A multiplex taqman real-time PCR for detection and differentiation of four antigenic types of canine parvovirus in China. Mol Cell Probes. (2018) 38:7–12. 10.1016/j.mcp.2018.02.004 29499233 PMC7126752

[8] Parthiban M Divya KC Kumanan K Bargavi DS. A rapid and highly reliable field-based lamp assay of canine parvovirus. Acta Virol. (2012) 56:71–4. 10.4149/av_2012_01_71 22404612

[9] Geng Y Wang J Liu L. Development of real-time recombinase polymerase amplification assay for rapid and sensitive detection of canine parvovirus. BMC Vet Res. (2017) 13:311. 10.1186/s12917-017-1232-z 29110666 PMC5674863

[10] Esfandiari J Klingeborn B. A comparative study of a new rapid and one-step test for the detection of parvovirus in faeces from dogs, cats and mink. J Vet Med. (2000) 47:145–52. 10.1046/j.1439-0450.2000.00328.x 10763385

[11] He J Wang Y Sun S Zhang X. Evaluation of chicken IgY generated against canine parvovirus viral-like particles and development of enzyme-linked immunosorbent assay and immunochromatographic assay for canine parvovirus detection. Viral Immunol. (2015) 28:489–94. 10.1089/vim.2015.0030 26469376

[12] Sharma C Singh M Upmanyu V Chander V Verma S Chakrovarty S. Development and evaluation of a gold nanoparticle-based immunochromatographic strip test for the detection of canine parvovirus. Arch Virol. (2018) 163:2359–68. 10.1007/s00705-018-3846-2 29736673

[13] Mochizuki M San G Nakatani H Yoshida M Harasawa R. Comparison of polymerase chain reaction with virus isolation and haemagglutination assays for the detection of canine parvoviruses in faecal specimens. Res Vet Sci. (1993) 55:60–3. 10.1016/0034-5288(93)90035-E 8397433

[14] Mukhopadhyay HK Amsaveni S Matta Sl Antony PX Thanislass J Pillai RM Development and evaluation of loop-mediated isothermal amplification assay for rapid and sensitive detection of canine parvovirus DNA directly in faecal specimens. Lett Appl Microbiol. (2012) 55:202–9. 10.1111/j.1472-765X.2012.03284.x 22748120 PMC7197762

[15] Liu W Dong D Yang Z Zou D Chen Z Yuan J. Polymerase Spiral Reaction (PSR): a novel isothermal nucleic acid amplification method. Sci Rep. (2015) 5:12723. 10.1038/srep12723 26220251 PMC4518254

[16] Gupta V Chakravarti S Chander V Majumder S Bhat SA Gupta VK. Polymerase Spiral Reaction (PSR): a novel, visual isothermal amplification method for detection of canine parvovirus 2 genomic DNA. Arch Virol. (2017) 162:1995–2001. 10.1007/s00705-017-3321-5 28349355

[17] Tanner NA Zhang Y Evans T. Visual detection of isothermal nucleic acid amplification using PH-sensitive dyes. Biotechniques. (2015) 58:59–68. 10.2144/000114253 25652028

[18] Wozniakowski G Fraczyk M Kowalczyk A Pomorska-Mól M Niemczuk K Pejsak Z. Polymerase Cross-Linking Spiral Reaction (PCLSR) for detection of African Swine Fever Virus (ASFV) in pigs and wild boars. Sci Rep. (2017) 7:42903. 10.1038/srep42903 28198455 PMC5309890

[19] Streck AF Rüster D Truyen U Homeier T. An updated taqman real-time PCR for canine and feline parvoviruses. J Virol Methods. (2013) 193:6–8. 10.1016/j.jviromet.2013.04.025 23680092 PMC7119788

[20] Mchugh Ml. Interrater reliability: the kappa statistic. Biochem Med. (2012) 22:276–82. 10.11613/BM.2012.031 23092060 PMC3900052

[21] Hoelzer K Shackelton LA Parrish CR Holmes EC. Phylogenetic analysis reveals the emergence, evolution and dispersal of carnivore parvoviruses. J Gen Virol. (2008) 89:2280–9. 10.1099/vir.0.2008/002055-0 18753238 PMC2735869

[22] Zhuang L Ji Y Tian P Wang K Kou C Gu N. Polymerase chain reaction combined with fluorescent lateral flow immunoassay based on magnetic purification for rapid detection of canine parvovirus 2. BMC Vet Res. (2019) 15:30. 10.1186/s12917-019-1774-3 30654823 PMC6337814

[23] Cao X Peng G Gu X He C Yue G Shi J Development and clinical evaluation of a direct amplification method to diagnose canine parvovirus and canine distemper viral infections in dogs without nucleic acid extraction. Pakistan J Zool. (2019) 51:1843–52. 10.17582/journal.pjz/2019.51.5.1843.1852

[24] Hoang M Wu HY Lien YX Chiou MT Lin CN. A Simpleprobe® Real-time PCR assay for differentiating the canine parvovirus type 2 genotype. J Clin Lab Anal. (2019) 33:e22654. 10.1002/jcla.22654 30168193 PMC6430354

[25] Wilkes RP Lee PY Tsai YL Tsai CF Chang HH Chang HF. An insulated isothermal PCR method on a field-deployable device for rapid and sensitive detection of canine parvovirus type 2 at points of need. J Virol Methods. (2015) 220:35–8. 10.1016/j.jviromet.2015.04.007 25889355 PMC7119629

[26] Liu L Wang J Geng Y Wang J Li R Shi R. Equipment-free recombinase polymerase amplification assay using body heat for visual and rapid point-of-need detection of canine parvovirus 2. Mol Cell Probes. (2018) 39:41–6. 10.1016/j.mcp.2018.04.004 29705183 PMC7127419

