# Supplementary figures and images for: An Improved Polymerase Cross-Linking Spiral Reaction Assay for Rapid Diagnostic of Canine Parvovirus 2 Infection
Source: Front Vet Sci. 2020 Oct 30;7:571629. doi: 10.3389/fvets.2020.571629 (PMC7661784; doi:10.3389/fvets.2020.571629)

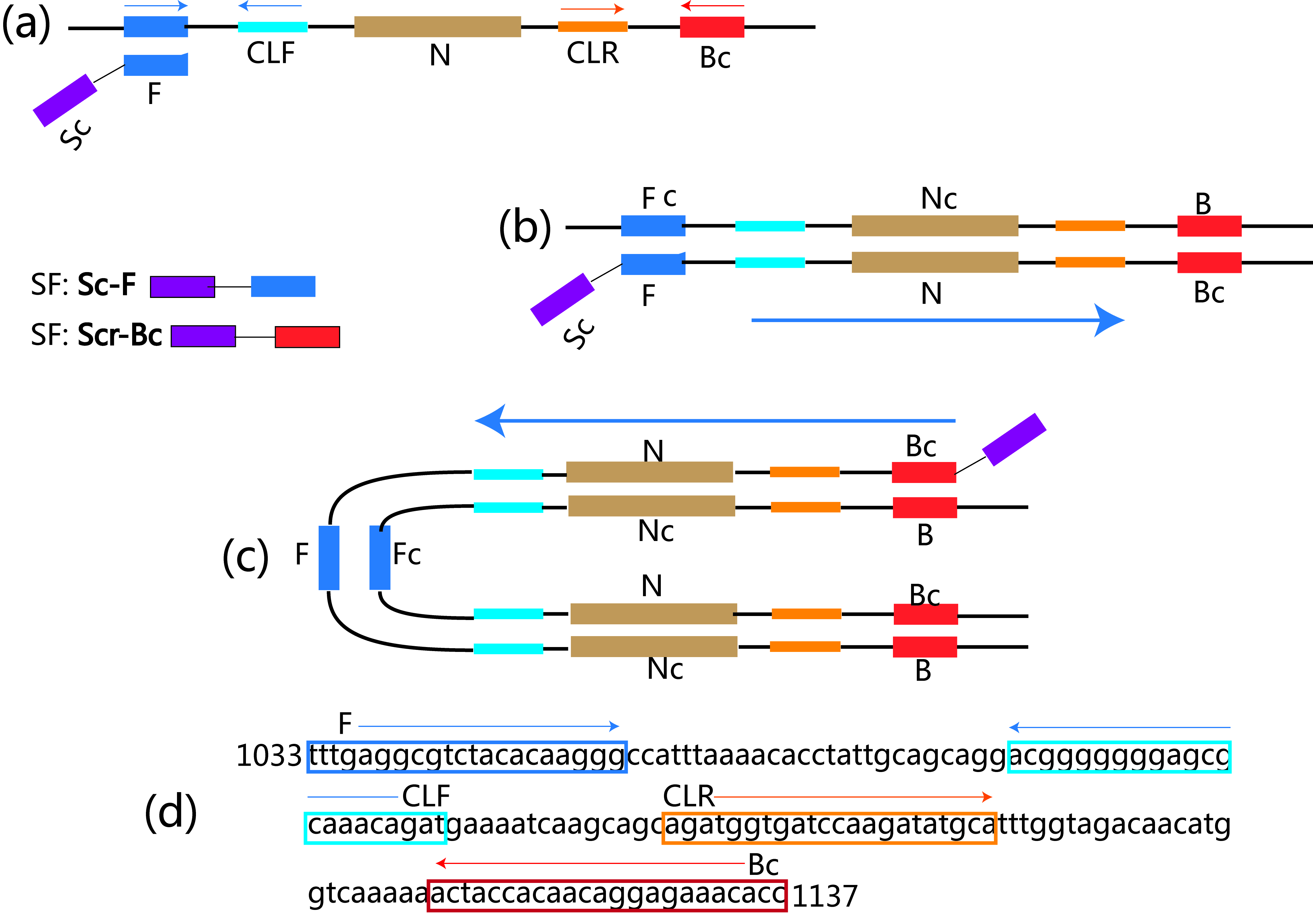

Supplement: Supplementary Figure 1 — (A–C) Schematic representation of the specific primers for PCLSR assay; (D) Schematic representation of the annealing positions of PCLSR primers within the CPV-2 VP2 gene. [file Image_1.TIF]
