# Supplementary material for: An Improved Polymerase Cross-Linking Spiral Reaction Assay for Rapid Diagnostic of Canine Parvovirus 2 Infection
Source: Front Vet Sci. 2020 Oct 30;7:571629. doi: 10.3389/fvets.2020.571629 (PMC7661784; doi:10.3389/fvets.2020.571629)
Supplement: Supplementary Table 1 — Assay data used for optimum reaction temperature and time for CPV-2 diagnosis by PCLSR. [file Table_1.DOCX]

Supplementary Table 1. Assay data used for optimum reaction temperature and time for CPV-2 diagnosis by PCLSR

| Time | Temperature | | | | | |
| --- | --- | --- | --- | --- | --- | --- |
|  | 60 °C | 61 °C | 62 °C | 63 °C | 64 °C | 65 °C |
| 30 min | + | + | + | + | + | + |
| 35 min | + | + | + | + | + | + |
| 40 min | + | + | + | + | + | + |
| 45 min | + | + | + | + | + | + |
| 50 min | + | ++ | ++ | ++ | + | + |
| 55 min | ++ | ++ | ++ | ++ | ++ | ++ |

+, ++: positive results obtained using PCLSR in three replicates on electrophoretic imaging and color change analysis
